# What is safe paravertebral block and neuraxial anesthesia for patients with neurofibromatosis type 1?

**DOI:** 10.1186/s40981-025-00798-5

**Published:** 2025-06-16

**Authors:** Hirotaka Kinoshita, Remi Kunii, Kishiko Nakai, Masato Kitayama, Junichi Saito

**Affiliations:** 1 Department of Anesthesiology, Hirosaki University Graduate School of Medicine, 5 Zaifu-Cho, Hirosaki, 036-8562 Japan; 2 Department of Operation, Hirosaki University Hospital, 53 Hon-Cho, Hirosaki, 036-8563 Japan

**To the Editor,**

Neurofibromatosis type 1 (NF1) is characterized by dermal neurofibromas and pigmentary lesions, including café-au-lait macules, skinfold freckling, and Lisch nodules [1]. Patients with NF1 may have spinal deformities and tumors; therefore, anesthesiologists must pay attention when administering neuraxial anesthesia. Herein, we report a case where a preoperative ultrasonic prescan for a thoracic paravertebral block (TPVB), performed before the resection of a giant mediastinal tumor, revealed multiple richly vascularized neurofibromas near the paravertebral space, leading to the cancellation of the TPVB.

A 16-year-old boy (height, 161 cm; weight, 36 kg) with NF1 was scheduled to undergo mediastinal tumor resection. At 15 years, an abnormal shadow was detected in his chest during a school health checkup. Chest computed tomography (CT) revealed an 18-cm schwannoma in his right posterior mediastinum, causing substantial compression of the right lung. Preoperative CT and magnetic resonance imaging (MRI) revealed no spinal or paravertebral neurofibromas (Fig. 1). Accordingly, total intravenous anesthesia combined with a TPVB was planned. After the induction of general anesthesia, we performed an ultrasonic prescan of the thoracic paravertebral space, which revealed multiple richly vascularized neurofibromas near the paravertebral space (Fig. 2). Some neurofibromas extended cranially beyond the level of the giant mediastinal tumors. Therefore, we canceled the TPVB and asked the surgeon to perform an intercostal nerve block under direct vision in the surgical field.

**Fig. 1:**
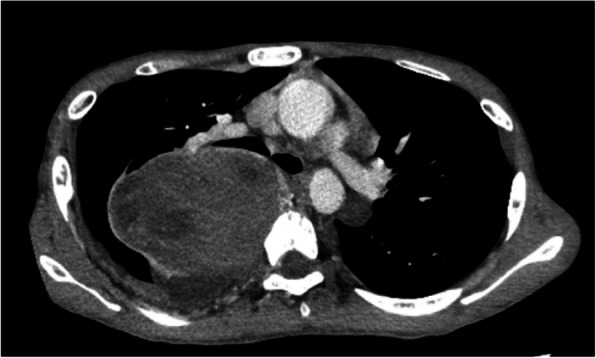
Preoperative computed tomography image at the level of the 5th thoracic vertebrae. Neurofibromas were not observed near the paravertebral space in this image

**Fig. 2:**
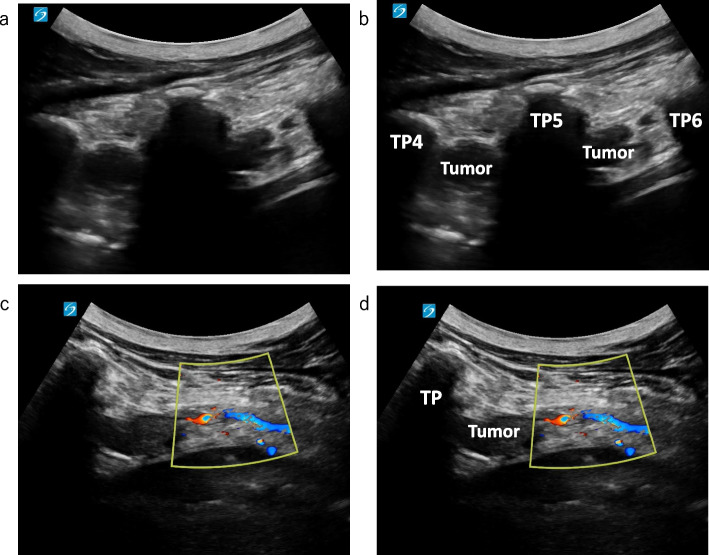
Ultrasonic prescan image of the thoracic paravertebral space. Sagittal view at the level of the 4–6th thoracic vertebrae (**a**, **b**) and Doppler ultrasound in transverse view at the level of the 5th thoracic vertebrae (**c**, **d**). Richly vascularized neurofibromas were observed near the paravertebral space. TP: transverse process. TP4, TP5, and TP6: transverse processes of the 4th, 5th, and 6th thoracic vertebrae, respectively

Avoiding neuraxial anesthesia in the absence of neuraxial imaging is recommended [2]. However, some researchers argue that preanesthetic neuraxial imaging may be unnecessary [3]. As demonstrated in this case, preoperative ultrasonic prescanning is useful for identifying neurofibromas that might not have been clearly delineated on CT or MRI. Neurofibromas are prone to bleeding, and plexiform neurofibromas or malignant transformations tend to be richly vascularized and carry a risk of dissemination [4]. Therefore, ultrasonography can play a crucial role in enhancing the safety of paravertebral block (PVB) and neuraxial anesthesia in patients with NF1. Furthermore, because neurofibromas tend to grow during pregnancy [5], performing preoperative ultrasonic prescans in patients with NF1 undergoing cesarean sections or painless deliveries may ensure the safe administration of neuraxial anesthesia during the perinatal period. Indeed, even in patients without visible skin tumors, as in this case, richly vascularized subcutaneous neurofibromatosis may still be present. Conducting a careful and deliberate prescan with adequate knowledge can help reduce the risk of overlooking important findings.

Sørenstua et al. used MRI to study the spread of local anesthetics to the intercostal space, paravertebral space, and neural foramina after an erector spine plane block in healthy volunteers [6]. In this case, there was a concern that the intravertebral neurofibromas might interfere with local anesthetic spread. Additionally, potential alterations in nerve conduction and the duration of blockade raised concerns regarding the adequacy of the anesthetic effect. Consequently, we asked the surgeon to perform an intercostal nerve block, considering its safety and anesthetic effects.

In conclusion, avoiding puncture of vascularized neurofibromas is important in regional anesthesia administration for NF1. Preoperative ultrasonic prescanning may enhance the safety of PVB and neuraxial anesthesia.

## Data Availability

Please contact the corresponding author for data requests.

## Abbreviations

CT: Computed tomography
MRI: Magnetic resonance imaging
NF1: Neurofibromatosis type 1
PVB: Paravertebral block
TPVB: Thoracic paravertebral block
